# Supplementary material for: Evidence that birds sleep in mid-flight
Source: Nat Commun. 2016 Aug 3;7:12468. doi: 10.1038/ncomms12468 (PMC4976198; doi:10.1038/ncomms12468)
Supplement: Supplementary Information — Supplementary Figures 1-19, Supplementary Table 1, Supplementary Discussion and Supplementary References [file ncomms12468-s1.pdf]

Supplementary figures

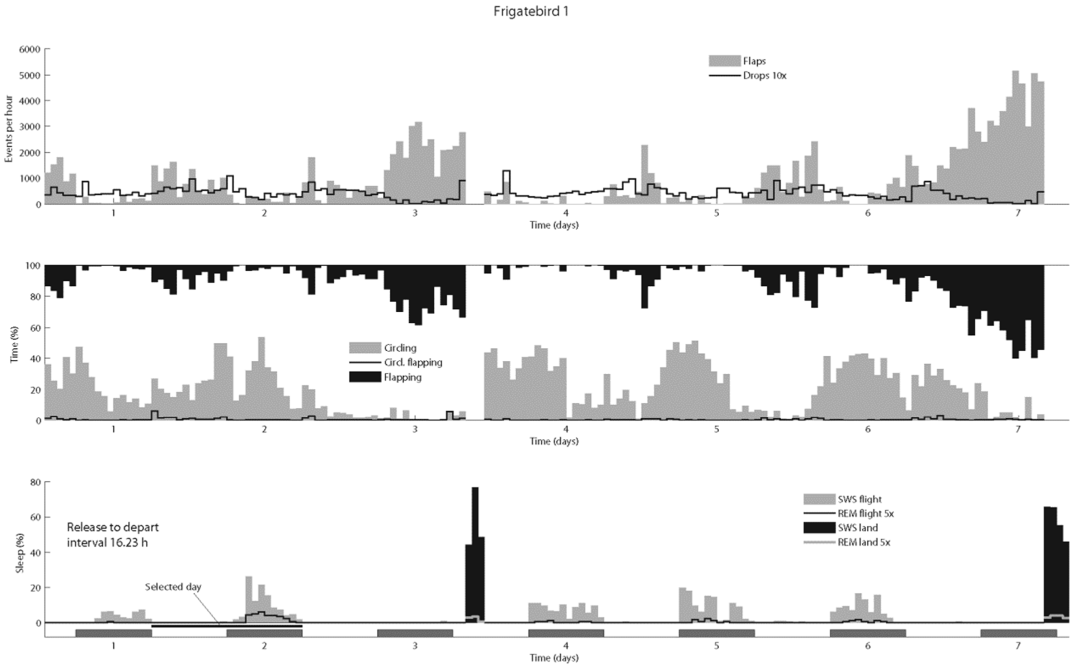

Supplementary Figure 1. Temporal distributions of flight behaviors and sleep across days for frigatebird 1. The top plot shows the number of wing flaps and drops. The middle plot shows the percentage of time spent flapping (without circling), and circling with and without flapping. The bottom plot shows the percentage of time spent in slow wave sleep (SWS) and rapid eye movement (REM) sleep. For all plots, data is shown for each hour beginning at the time when the birds initiated their first flight after instrumentation and ending at the time of recapture. The time interval from release to initiating flight in noted at the left of the bottom plot. Periods with all flight parameters at zero correspond to times when the birds were on land. Night (sunset to sunrise) is marked with grey bars below the x-axis of the bottom plot. The day with large amounts of sleep selected for a detailed analysis is marked by a black horizontal line segment. Drops and REM sleep were multiplied 10 and 5 times, respectively, to enhance visualization.

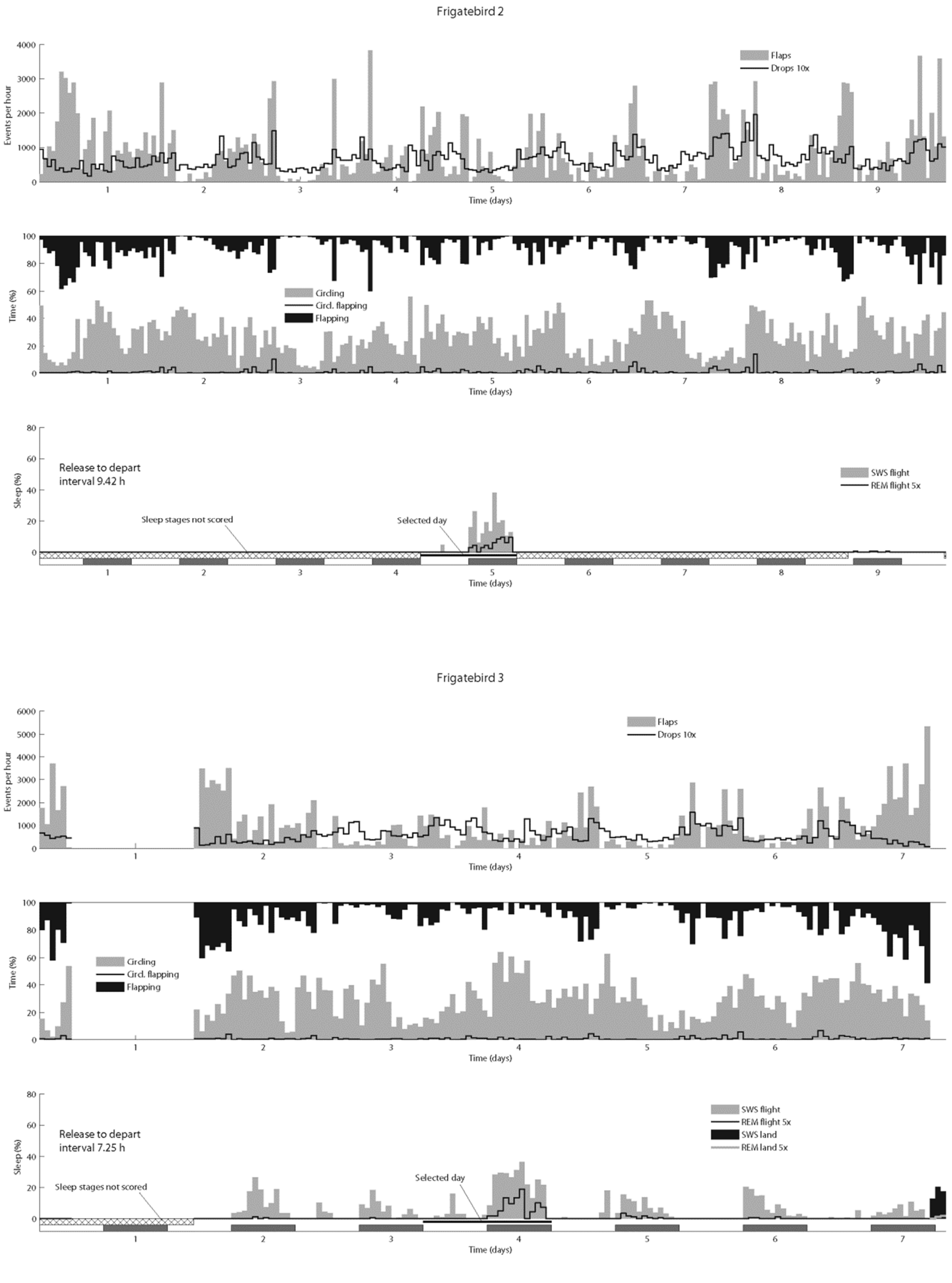

Supplementary Figure 2. Temporal distributions of flight behaviors and sleep across days for frigatebirds 2 and 3. Data plotted as in Supplementary Fig. 1. Data excluded from the analysis is marked by the cross-hatched bar above the photoperiod.

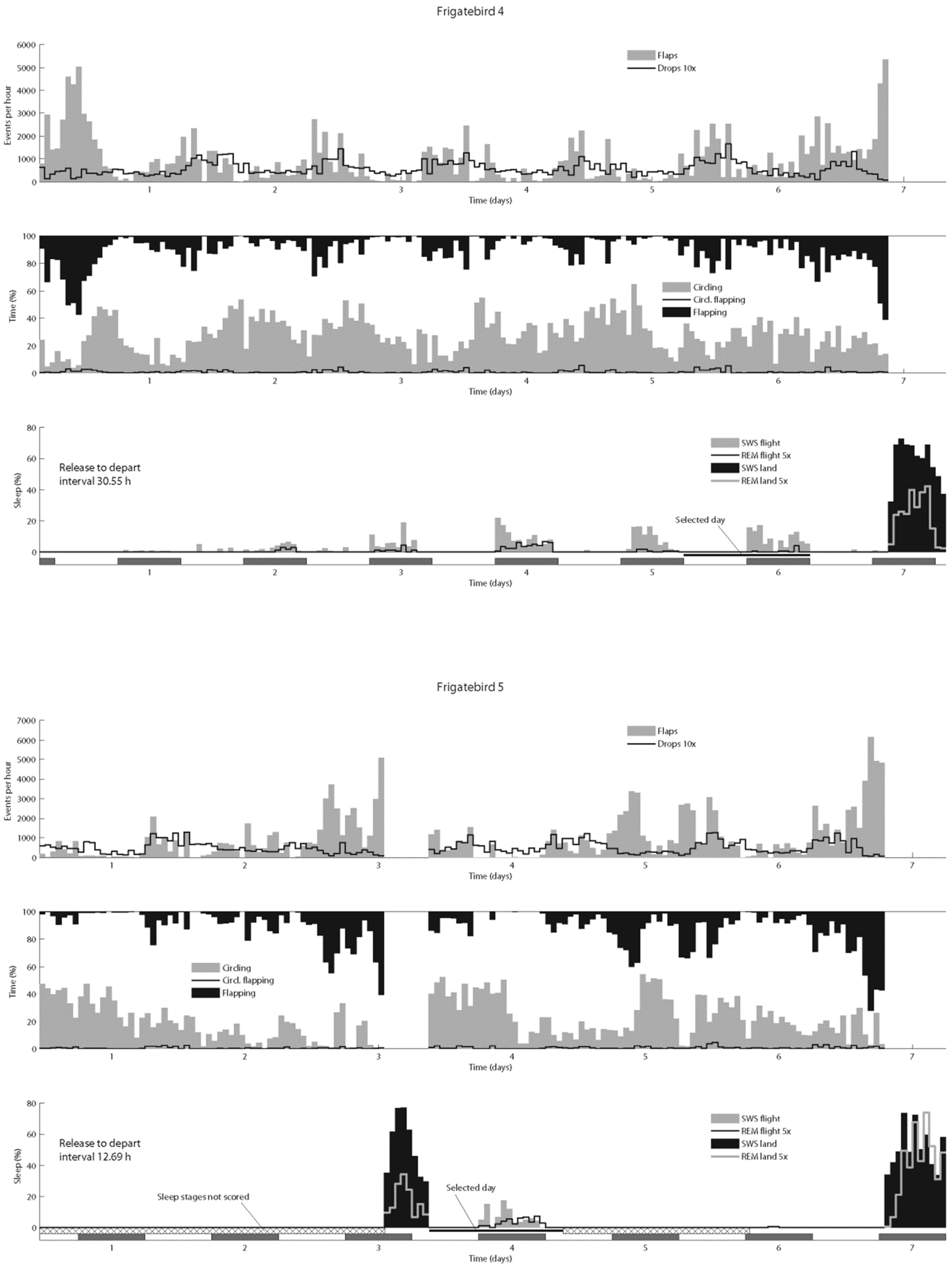

Supplementary Figure 3. Temporal distributions of flight behaviors and sleep across days for frigatebirds 4 and 5. Data plotted as in Supplementary Fig. 1. Data excluded from the analysis is marked by the cross-hatched bar above the photoperiod.

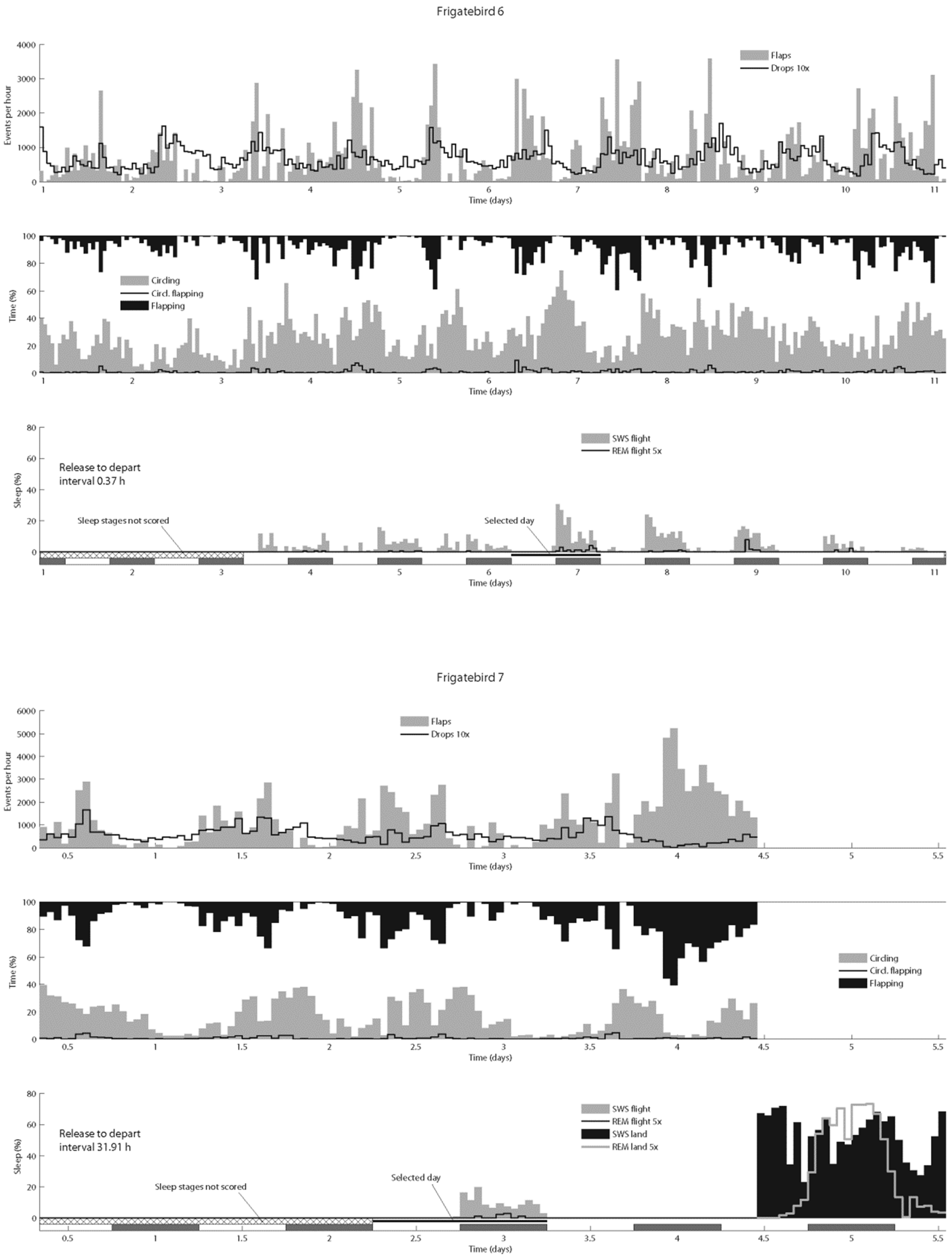

Supplementary Figure 4. Temporal distributions of flight behaviors and sleep across days for frigatebirds 6 and 7. Data plotted as in Supplementary Fig. 1. Data excluded from the analysis is marked by the cross-hatched bar above the photoperiod.

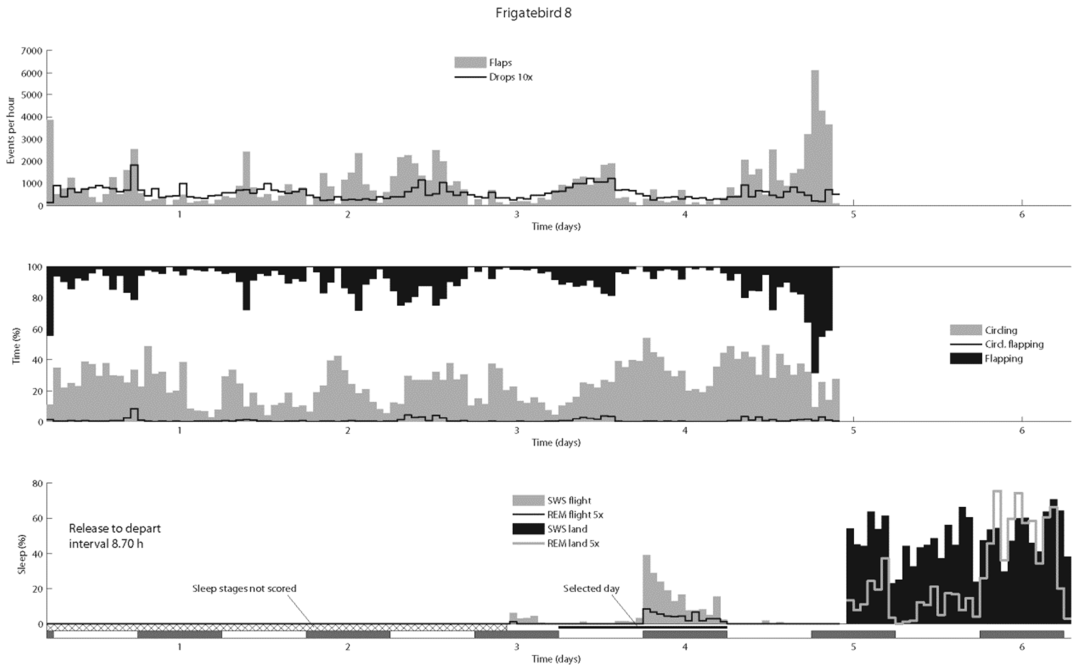

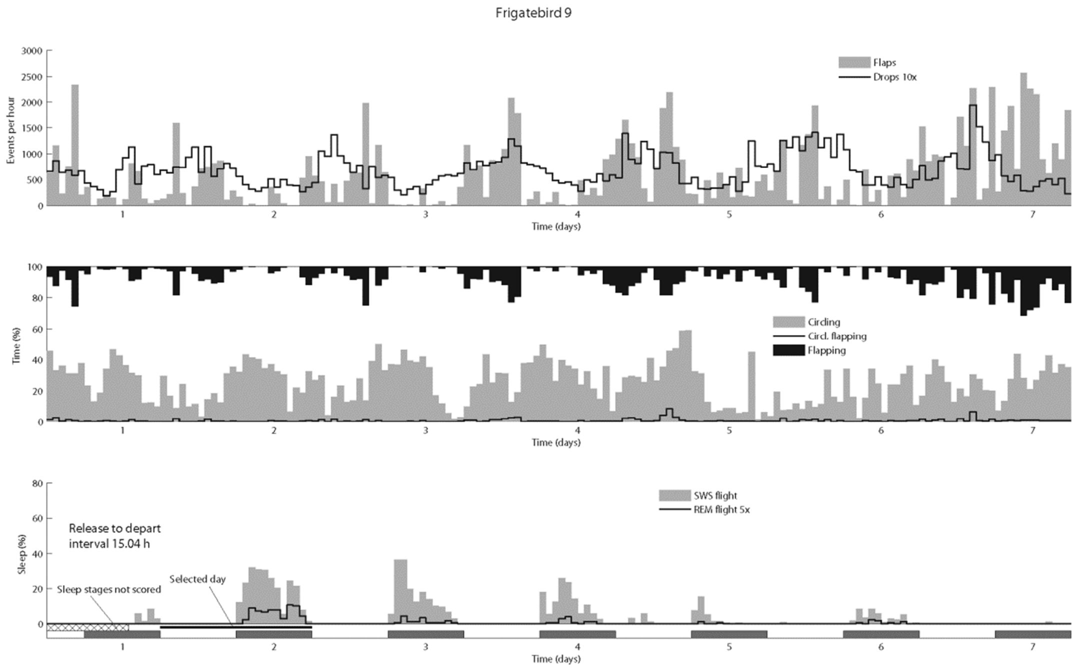

Supplementary Figure 5. Temporal distributions of flight behaviors and sleep across days for frigatebirds 8 and 9. Data plotted as in Supplementary Fig. 1. Data excluded from the analysis is marked by the cross-hatched bar above the photoperiod.

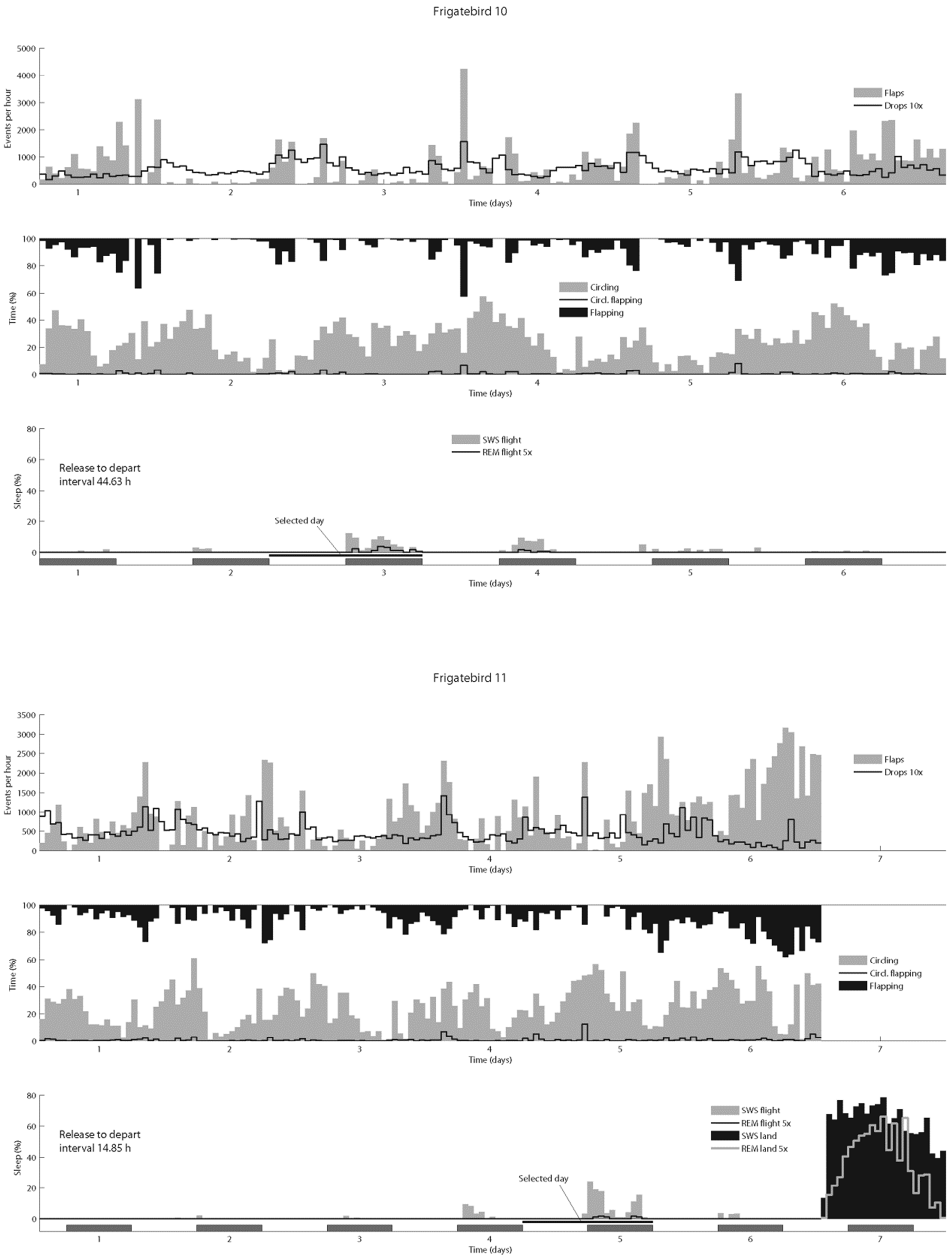

Supplementary Figure 6. Temporal distributions of flight behaviors and sleep across days for frigatebirds 10 and 11. Data plotted as in Supplementary Fig. 1.

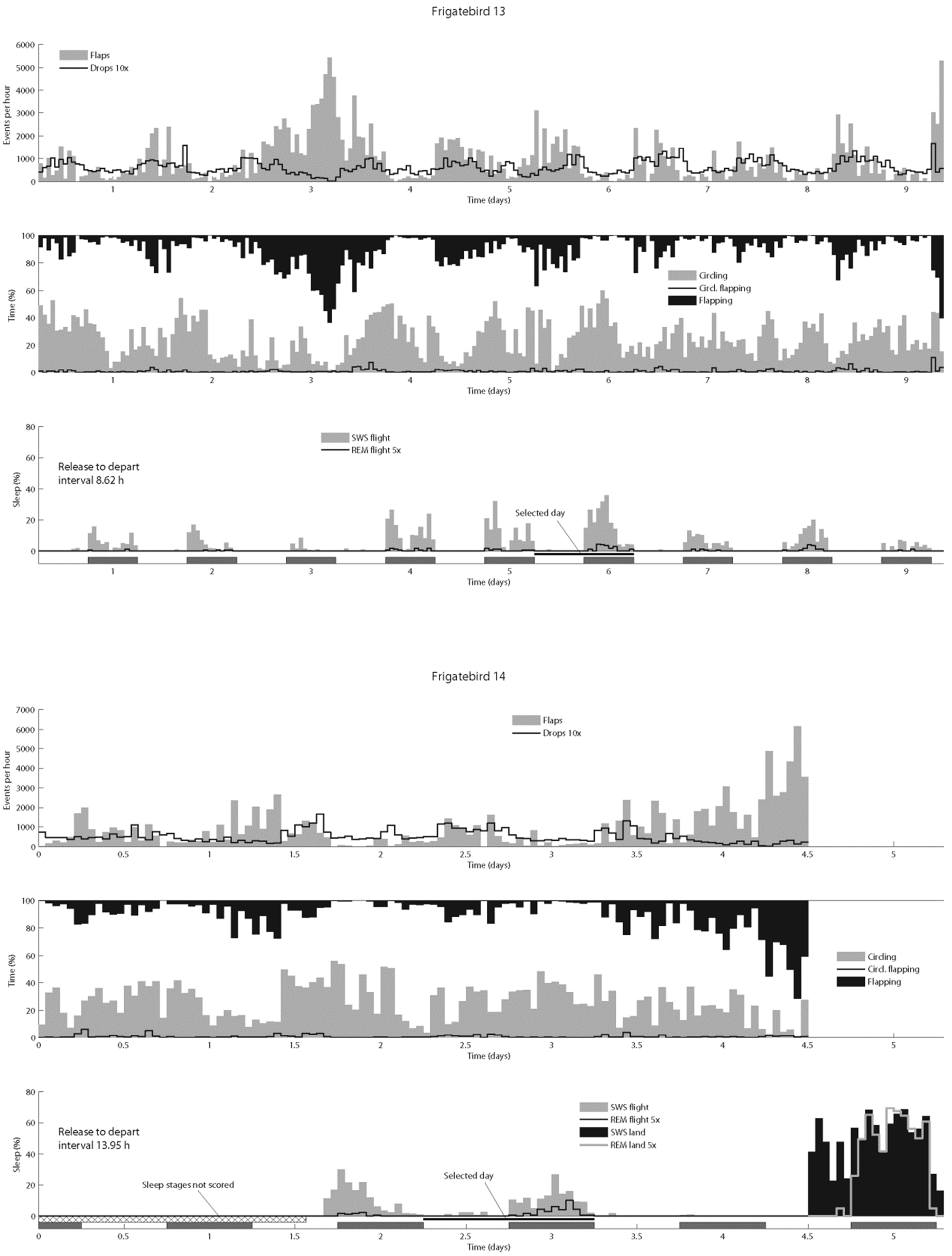

Supplementary Figure 7. Temporal distributions of flight behaviors and sleep across days for frigatebirds 13 and 14. Data plotted as in Supplementary Fig. 1. Data excluded from the analysis is marked by the cross-hatched bar above the photoperiod.

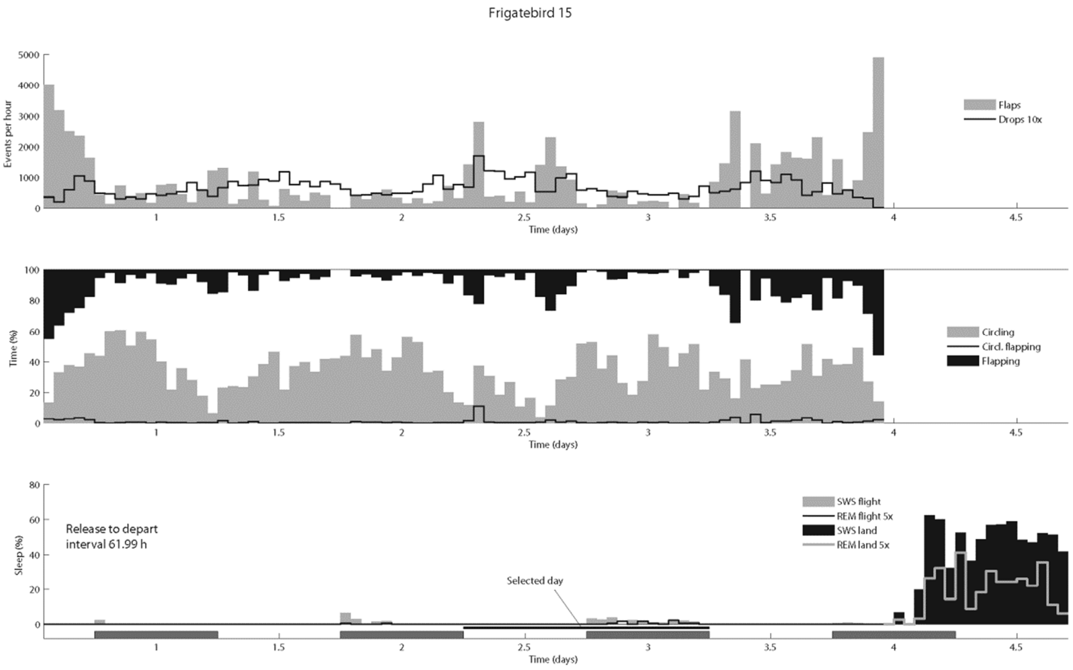

Supplementary Figure 8. Temporal distributions of flight behaviors and sleep across days for frigatebird 15. Data plotted as in Supplementary Fig. 1.

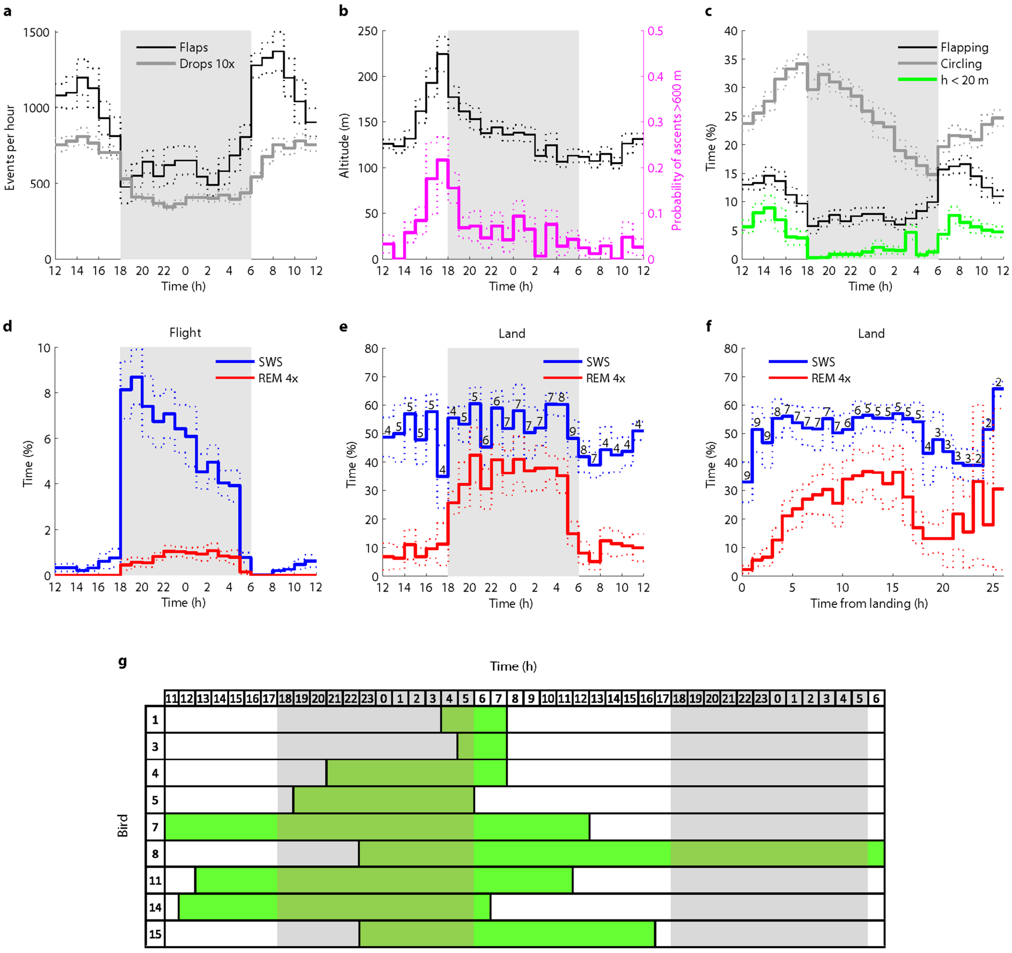

Supplementary Figure 9. Average hourly distribution of flight behaviors and sleep in frigatebirds. Unless specified otherwise, the values reflect the mean (s.e.m., dotted line) of the hourly means for individual birds (N=14). (a) Distribution of wing flaps and drops. Note the reduced number of wing flaps and drops at night marked by grey. (b) Hourly flight altitude calculated from the GPS data. The secondary y-axis shows the probability of a high-altitude ascent (>600 m) occurring during a given hour. (c) Percent of time spent in flapping flight, circling, and flying at low altitude (<20 m). The gradual decrease and increase in circling time across the night and day, respectively, likely reflects the availability of thermals (significance of linear trends, P=1.8×10^{-7} and P=2.4×10^{-6}). Flying at low altitude, a behavior usually associated with feeding, was more prevalent during the day than the night (P=3.9×10^{-5}, paired two-tailed Student's t-test). (d) Percent of time spent in slow wave sleep (SWS) and rapid eye movement (REM) sleep in flight (N=14, average of all days). Data for REM sleep was multiplied 4 times to enhance visualization. Note that the percentages of SWS and circling flight decline in parallel across the night (P=5.6×10^{-6} for the linear trend for the entire night, and P=1.2×10^{-6} with the last hour of the night excluded); however, the decline in SWS in individual birds does not correlate with the decline in circling (Pearson correlation coefficient R=-0.090, P=0.76). (e) SWS and REM sleep on land aligned to the light cycle. In (e) and (f), the number of birds with data for a given hour is indicated above the data for that hour. (f) SWS and REM sleep on land aligned to the time from landing. (g) Chart showing the recording period following the final landing for each bird (green) rounded to the nearest hour. For birds with two data points for an hour, the average was used in (e). For birds with recordings lasting >24 h, only the first 24 were used in (f). For birds with two landings (1 and 5), only data for the final landing was used in (e) and (f).

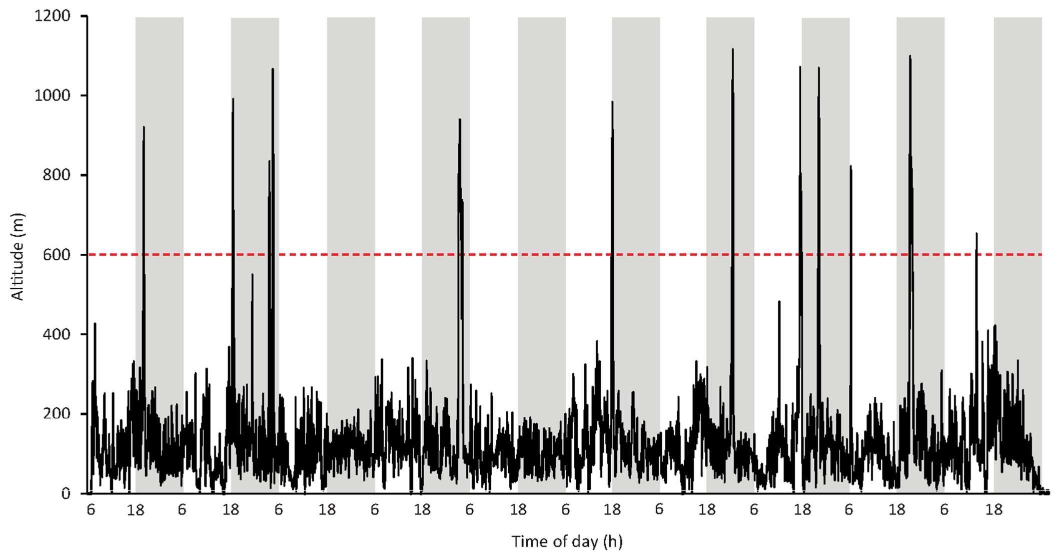

Supplementary Figure 10. Altitude across a 10 day flight for frigatebird 2. Note the occasional ascents above 600 m (dashed red line). Nights are indicated by the grey boxes.

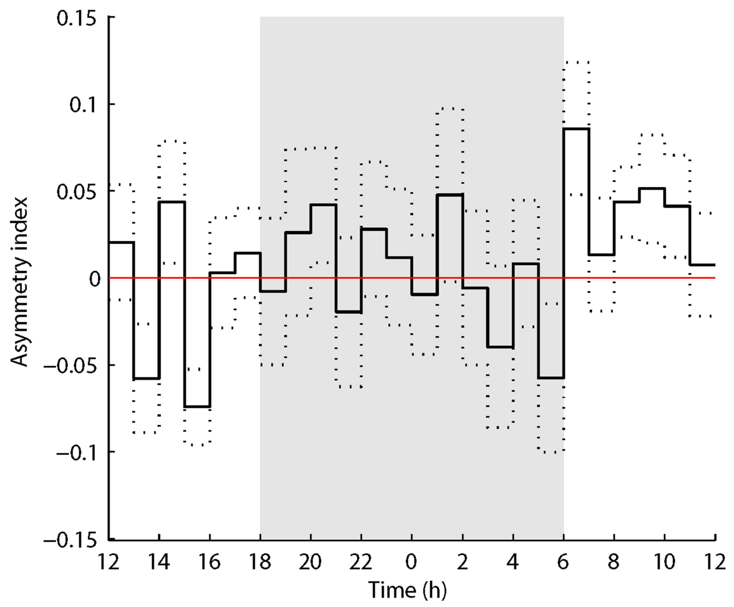

Supplementary Figure 11. Asymmetry index showing the absence of a left-right bias in circling behavior. Asymmetry index AI=(L-R)/(L+R), L – time circling to the left, R – time circling to the right. The values reflect the mean (s.e.m., dotted line) of the hourly means for individual birds (*N*=14). The AI averaged across all hours does not does not differ from zero (AI=0.0055±0.0169, mean±s.e.m., *P*=0.75; two-tailed Student's *t*-test).

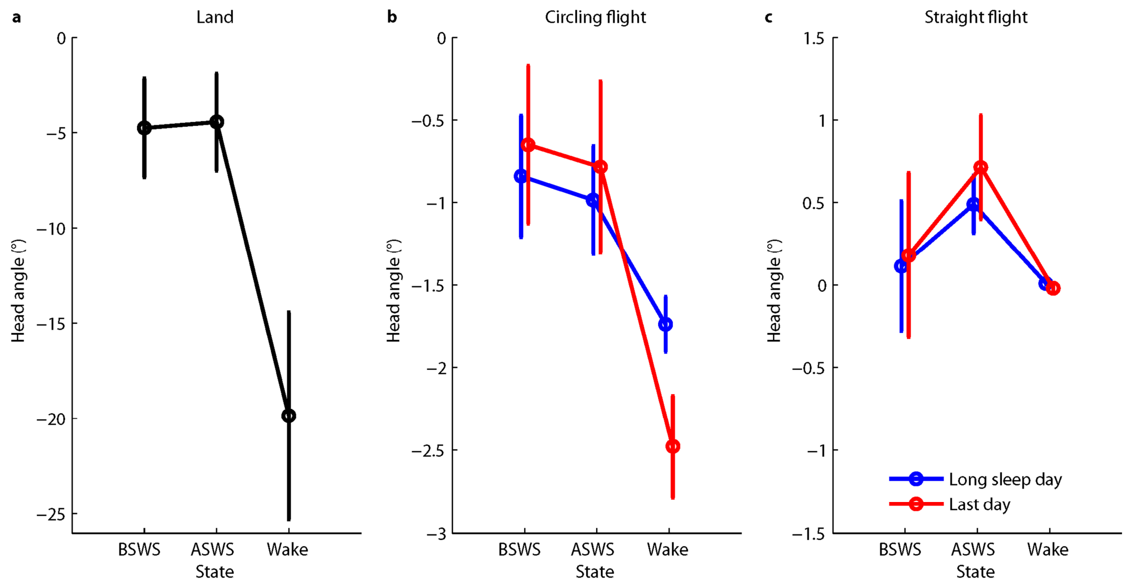

Supplementary Figure 12. Relationships between head angle and brain state during flight and while on land. Head angle relative to the horizon while awake and during bihemispheric and asymmetric slow wave sleep (BSWS and ASWS, respectively) while on land (a) and during circling (b) and straight (c) flight. Blue lines show head angles during a day selected for large amounts of sleep in flight (see Supplementary Fig. 1-8), and red lines show head angle during last day of flight, when a relatively small amount of sleep was detected. Angles were calculated from the average head position in straight flight taken as zero. On land (N=9), the head angled down more during wakefulness than during BSWS (P=0.008; a paired two-tailed Student's t-test was used here and below) and ASWS (P=0.008), likely reflecting a combination of changes in head posture related to brain state and attending to the chick during wakefulness. During circling flight (N=14), the head also angled down more during wakefulness than BSWS (P=0.0032 on long sleep day, $P=3.3\times10^{-5}$ on the last day, and $P=1.4\times10^{-5}$ for both days taken together) and ASWS (P=0.0036, $P=9.2\times10^{-5}$, and $P=5.0\times10^{-6}$). During BSWS the beak was held higher than during ASWS on day with long sleep (P=0.031), but not on the last day (P=0.53) or both days pooled together (P=0.16). Nonetheless, the sleeping and waking head angles and the magnitude of their difference were smaller during circling flight than on land. The smaller angular deviations likely reflect the absence of attending to the chick in flight, as well as overall differences in head position relative to the body during flight when compared to land. In this regard, the difference in head angle between sleep and wakefulness during circling flight is the most informative. The relationship between head angle and brain state during straight flight had similar tendencies, but was more variable than during circling flight; only ASWS had a significantly (P=0.017, P=0.042, and P=0.0014) higher head angle than wakefulness. The absence of a significant difference between the head angle during BSWS and wakefulness (P=0.79, P=0.73, and P=0.53) in straight flight might be linked to larger aerodynamic disturbances produced by the higher airspeed, shorter sleep episodes and somewhat lighter sleep. In straight flight, slow wave activity (SWA; 0.75–4.5 Hz power) during SWS was slightly lower than in circling flight (280±23 μV^2 vs. 294±28 μV^2, 4.6% difference, P=0.31) and gamma power was significantly higher (13.4±2.0 μV^2 vs. 11.5±1.7 μV^2, 16.4% difference, P=0.0014). SWA and gamma power during SWS were computed for long sleep day only.

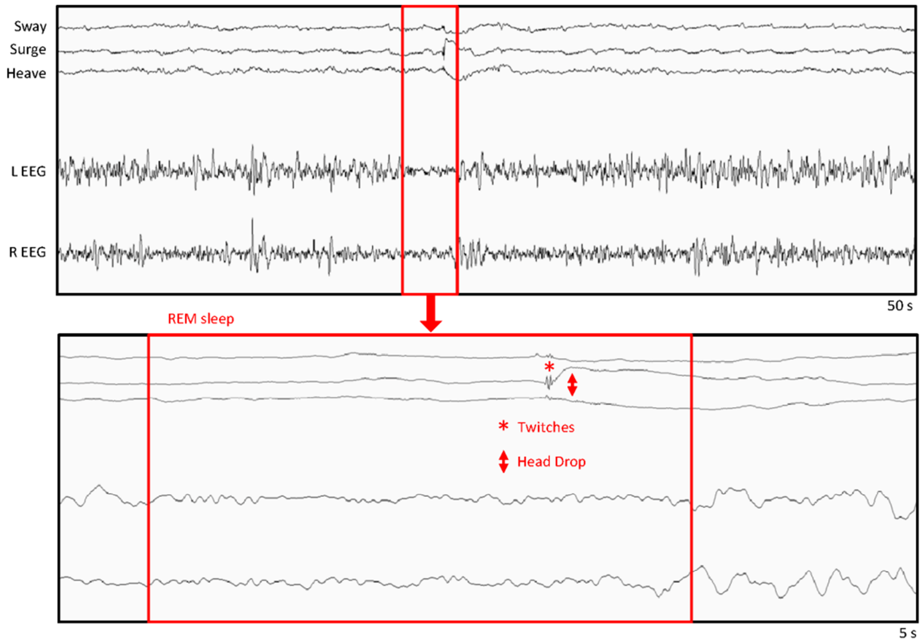

Supplementary Figure 13. REM sleep in flight. Expanded view (5 s) of the episode of REM sleep (red box) marked by the red arrow in Fig. 2a. REM sleep was characterized by low-amplitude, high-frequency electroencephalogram (EEG) activity in both hemispheres, head dropping (red arrow), and occasional twitches (*).

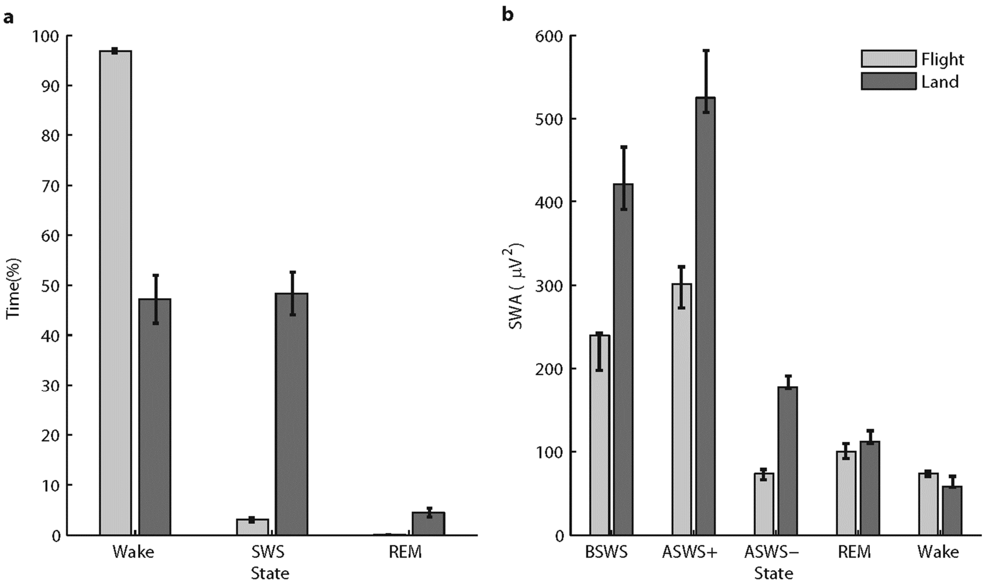

Supplementary Figure 14. Frigatebirds sleep more and deeper on land than in flight. This figure is analogous to Fig. 4a, b, but for all 14 birds recorded in flight and, as before, the 9 birds also recorded on land. (a) Time spent awake and in slow wave sleep (SWS) and rapid eye movement (REM) sleep in flight and on land. (b) Electroencephalogram (EEG) slow wave activity (SWA; 0.75–4.5 Hz power; median and quartiles are plotted) while awake and during SWS and REM sleep in flight and on land. For SWS, SWA is shown for, 1) bihemispheric SWS (BSWS), 2) asymmetric SWS (ASWS) for the hemisphere with greater SWA (ASWS+), and 3) ASWS for the hemisphere with lower SWA (ASWS–).

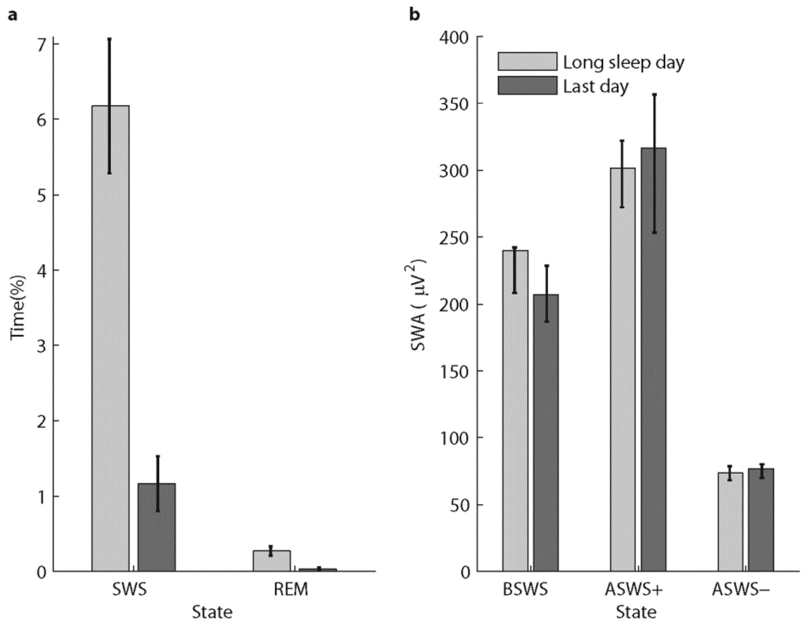

Supplementary Figure 15. Sleep depth does not differ on days with large and small amount of sleep. (a) On the day with large amounts of sleep (see Supplementary Fig. 1-8), the times spent in slow wave sleep (SWS) and rapid eye movement (REM) sleep were higher than on the last day of flight (P=4.7×10^{-5} and 0.0011, respectively; N=14, mean±s.e.m.; a paired two-tailed Student's *t*-test was used here and below). (b) SWS depth estimated by electroencephalogram (EEG) slow wave activity (SWA; 0.75–4.5 Hz power) did not differ between these two days for any state (P=0.28, P=0.57 and P=0.067; median and quartiles are plotted). For SWS, SWA is shown for, 1) bihemispheric SWS (BSWS), 2) asymmetric SWS (ASWS) for the hemisphere with greater SWA (ASWS+), and 3) ASWS for the hemisphere with lower SWA (ASWS–).

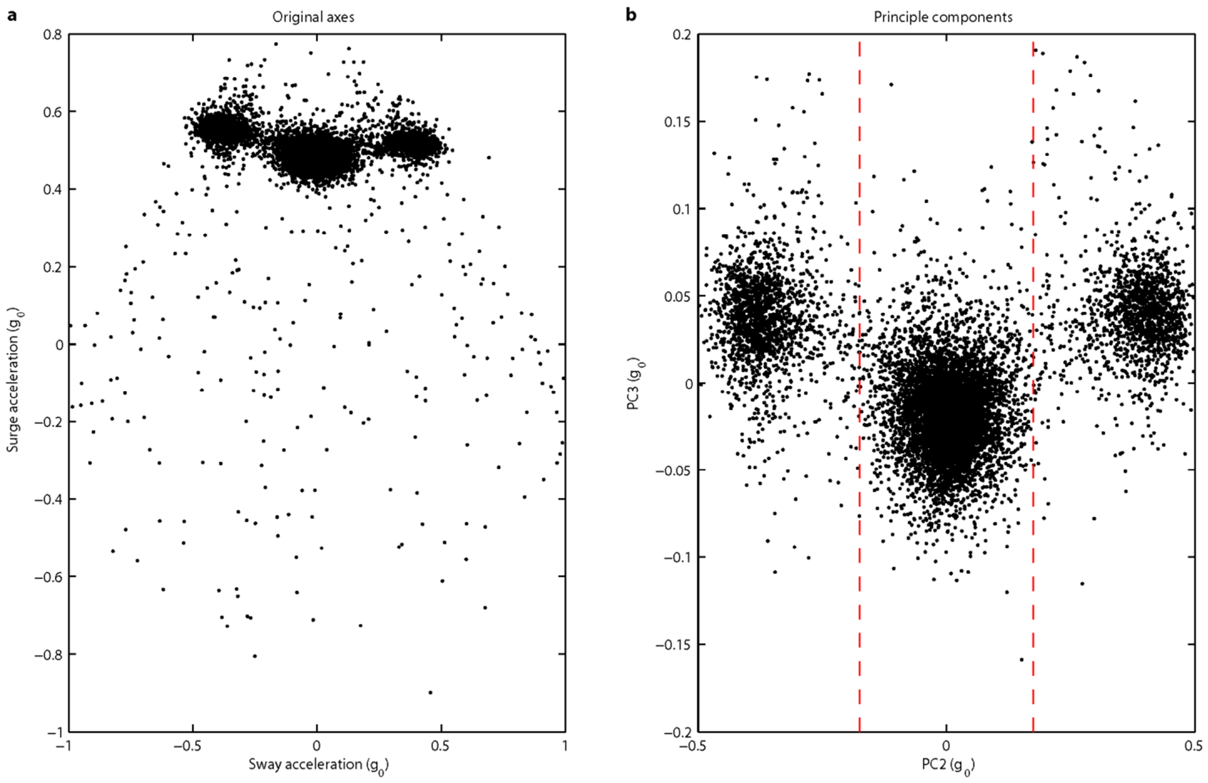

Supplementary Figure 16. Distribution of the total acceleration vector in the sway-surge plane. (a) Distribution of total acceleration in the plane of the two original accelerometer axes lying in a plane tangential to the skull. A small asymmetry in acceleration was caused by the slightly asymmetrical attachment of the accelerometer to the birds' head. This asymmetry was compensated for by rotating the coordinate axes in the direction of principle components (PCs). (b) The same as (a) but in the plane of the 2nd and the 3rd PCs. Dots represent 0.1 Hz low-pass filtered acceleration taken at 4-s intervals from one bird. The three clearly visible clusters correspond to the three modes of flight: straight (center cluster), and circling with rotation to the left and to the right (right and left clusters, respectively). Red dotted lines at ±0.175 g_0 show borders separating straight from circling flight.

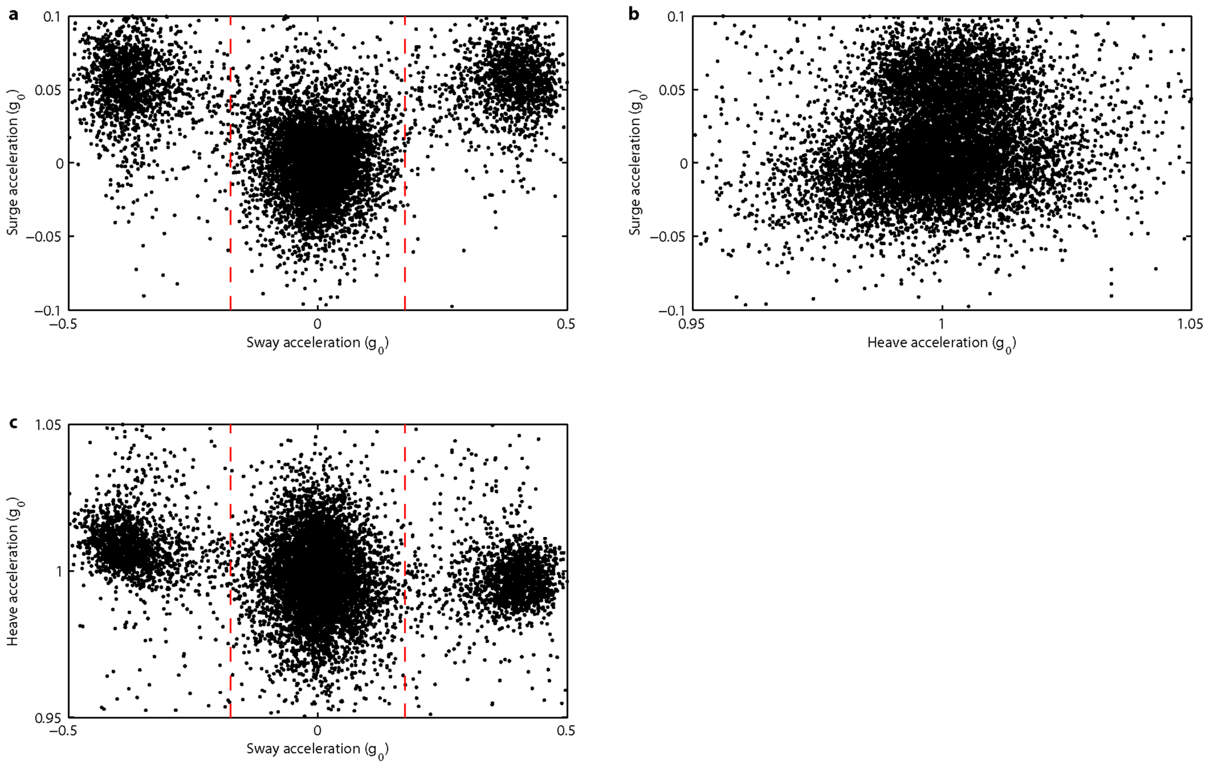

Supplementary Figure 17. Distribution of the total acceleration vector in the three orthogonal planes with the coordinate system zeroed in the center of the straight flight cluster. (a) Plane sway - surge. (b) Plane heave - surge. (c) Plane sway - heave. Centers of the side clusters were taken as proxies for the coordinates of the acceleration in circling flight (see Supplementary Table 1). These coordinates were used to compute the head turn in circling flight.

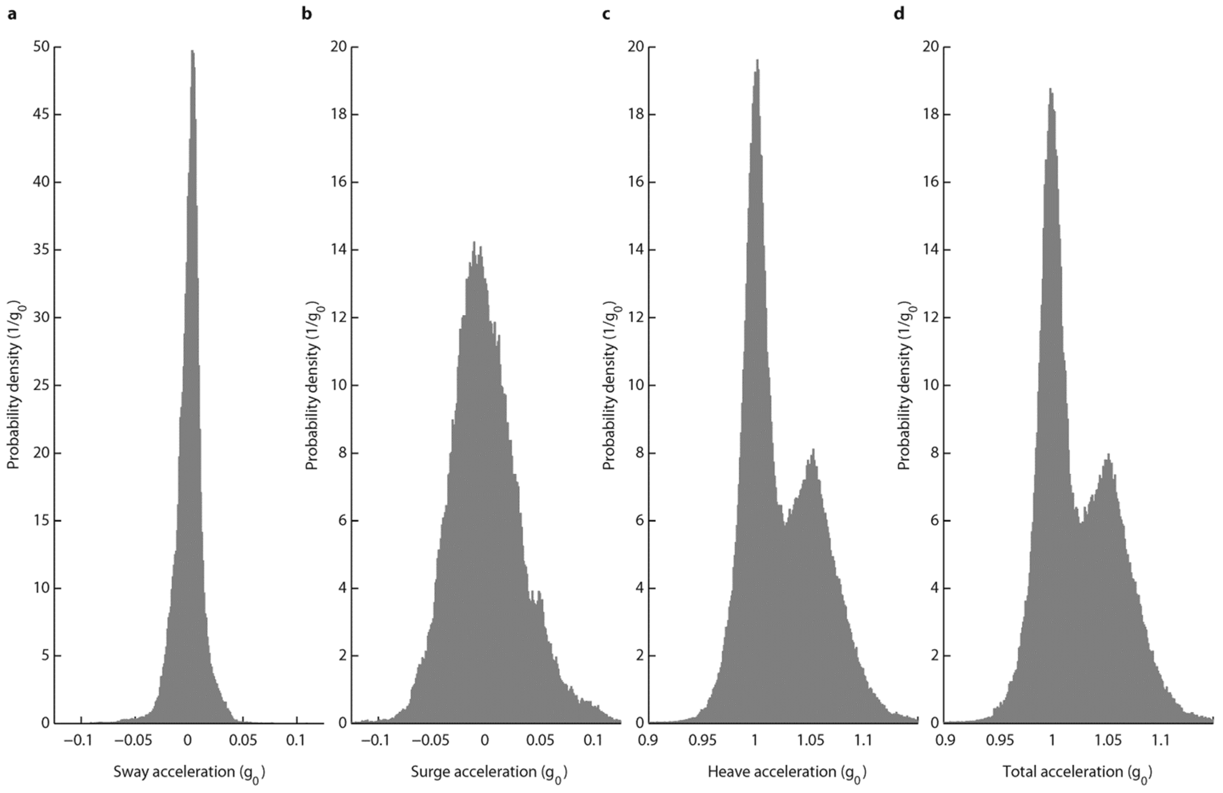

Supplementary Figure 18. Distribution of accelerations recorded with a back-mounted accelerometer in flight. (a) Sway acceleration. (b) Surge acceleration. (c) Heave acceleration. (d) Total acceleration. Similar to the head-fixed accelerometer, the axes were rotated to have one directed toward the ground, and another in the lateral direction. The total duration of flight used for plotting the histograms was 20.4 h. The accelerometer sampling rate was 25 Hz and the bin width was 0.001 g_0. Note that the distributions of heave and total accelerations are bi-modal. The two modes reflect straight (peak at 1 g_0) and circling (peak at 1.052 g_0) flights. Opposite to heave, the sway and surge accelerations distributions are uni-modal and the sway distribution is especially narrow. The latter indicates that the lateral deviation of the vector of total acceleration is very small in both straight and circling flight. Consequently, although an accelerometer placed on the back can discriminate circling from straight flight modes, only the head-mounted accelerometer can be used to determine the direction of rotation.

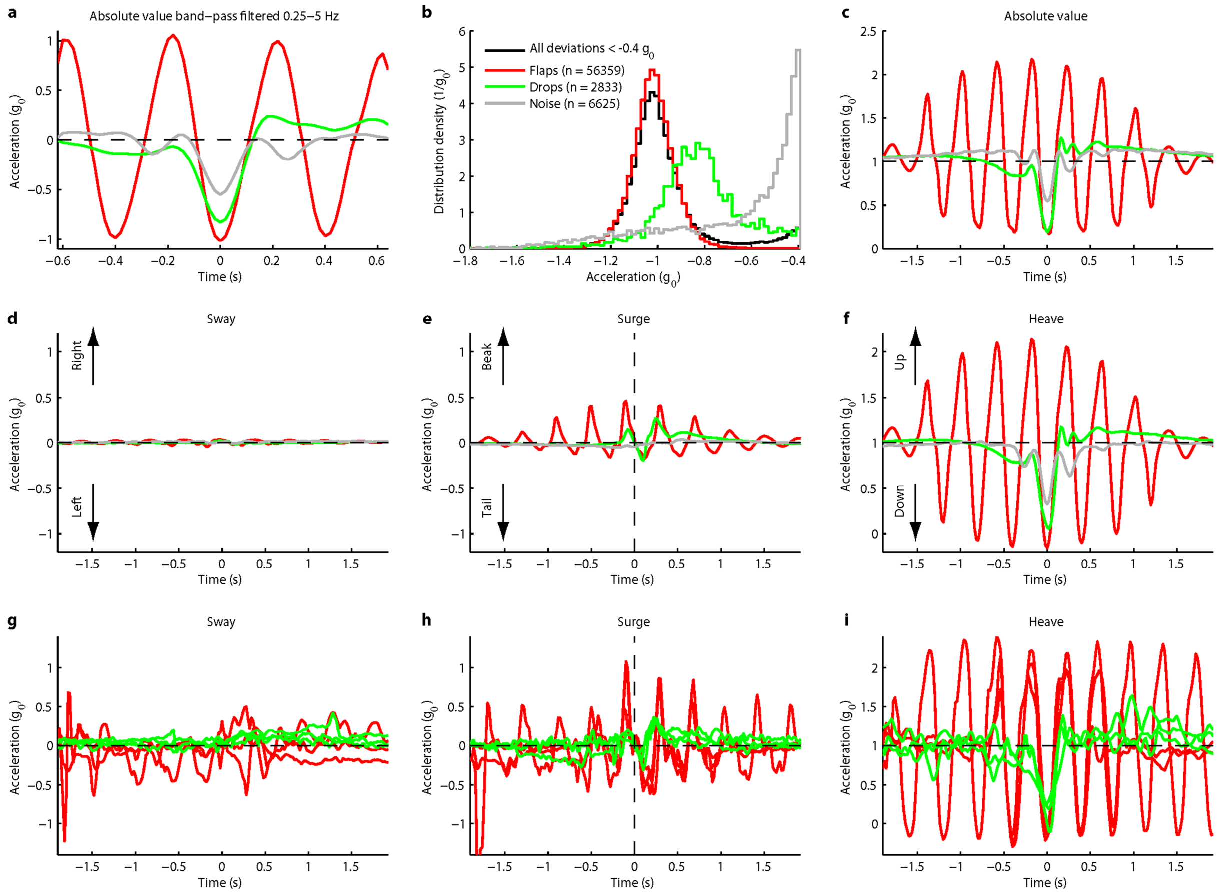

Supplementary Figure 19. Acceleration due to wing flaps and drops. This chart shows data from frigatebird 1 (night selected for large amounts of sleep marked in Supplementary Fig. 1). (a) Averaged shape of 0.25–5 Hz filtered total acceleration around flaps/drops detection points. Note symmetrical form of flaps and asymmetrical shape of drops. (b) Distribution density of maximal deviation (at time zero) for flaps, drops, and noise. Note that flaps can be easily separated from noise by selecting a threshold around -0.6 g_0. Separation of drops from flaps and noise requires additional information about the signal shape. (c) Absolute value of acceleration as in (a), but without filtration. (d-f) Acceleration in the three orthogonal directions, sway, surge, and heave. Note that the acceleration curves in the surge (tail-beak) direction are shifted upwards relative to the zero level for both flaps and drops. This means that drops are not only used to change direction, as one could suppose, but also to increase flight speed by converting potential energy of the bird into kinetic energy. Acceleration in the surge and heave directions are phase shifted by approximately 90°. (g-i) Raw recordings of acceleration during three randomly selected flaps and three randomly selected drops.

Supplementary tables

Supplementary Table 1. Acceleration-related flight parameters of individual birds. Skull angle represents the deviation of the skull tangential plane from the horizon in straight flight. All other parameters relate to circling flight (see Methods for details). Bank angle and head turn angle (computed from the position in straight flight) significantly differ from zero in circling flight (P=6.4×10^{-15} and P=3.1×10^{-8}, respectively; two-tailed Student's *t*-test). However, these parameters do not correlate with each other (Pearson correlation coefficient R=-0.012, P=0.69). The head angle deviations in the right-left and beak-tail directions are also significant (P=0.001 and P=3.2×10^{-5}, respectively; two-tailed Student's *t*-test). Index L indicates that the sign of the marked parameters refers to circling flight with turning to the left. For right turns, the signs of these parameters should be opposite; given the left-right symmetry, common numeric values are given for left/right turns.

		Acceleration in head-fixed coordinate system (g_0)						Head rotation axis (coordinates of unit vector) and angle				Head angular deviation (°)	
**Animal**	**Skull angle (°)**	**$Sway_L$**	**Surge**	**Heave**	**Total acceleration (g_0)**	**Radial acceleration (g_0)**	**Bank angle (°)**	**Right-$left_L$**	**Beak-tail**	**Down-up**	**Head turn (°)**	**Right-$left_L$**	**Beak-tail**
1	29.614	0.309	0.044	1.009	1.056	0.339	18.722	0.777	0.572	-0.263	2.919	1.653	-2.279
2	26.837	0.362	0.024	1.005	1.069	0.378	20.704	0.768	0.571	-0.290	1.555	0.884	-1.197
3	28.524	0.312	0.038	1.011	1.059	0.348	19.204	0.662	0.713	-0.231	2.909	2.064	-1.937
4	30.121	0.355	0.023	1.008	1.069	0.376	20.627	0.653	0.716	-0.246	1.730	1.235	-1.134
5	32.587	0.283	0.039	1.003	1.043	0.297	16.555	0.901	0.340	-0.268	2.296	0.770	-2.073
6	30.340	0.345	-0.011	1.007	1.065	0.366	20.082	-0.428	0.890	0.156	1.289	1.147	0.554
7	35.291	0.297	0.034	1.010	1.053	0.330	18.273	0.667	0.712	-0.220	2.607	1.847	-1.747
8	26.014	0.354	0.004	1.009	1.069	0.378	20.684	0.147	0.988	-0.055	1.368	1.351	-0.201
9	31.039	0.310	0.014	1.003	1.050	0.319	17.710	0.785	0.566	-0.251	0.918	0.518	-0.722
10	27.923	0.298	0.031	1.011	1.055	0.336	18.558	0.594	0.779	-0.200	2.725	2.115	-1.629
11	33.251	0.292	0.027	1.007	1.049	0.316	17.554	0.694	0.686	-0.219	2.007	1.371	-1.397
13	29.361	0.300	0.031	0.991	1.036	0.271	15.185	0.701	-0.688	-0.190	2.389	-1.649	-1.667
14	29.025	0.374	0.056	1.004	1.073	0.388	21.229	0.900	0.259	-0.350	3.121	0.782	-2.818
15	28.076	0.308	0.038	1.001	1.048	0.315	17.472	0.938	0.183	-0.295	2.090	0.373	-1.962
**Mean**	**29.857**	**0.321**	**0.028**	**1.006**	**1.057**	**0.340**	**18.754**	**0.626**	**0.521**	**-0.209**	**2.137**	**1.033**	**-1.444**
STD	2.539	0.030	0.017	0.005	0.011	0.035	1.779	0.359	0.415	0.125	0.688	0.945	0.872
**SEM**	**0.678**	**0.008**	**0.005**	**0.001**	**0.003**	**0.009**	**0.475**	**0.096**	**0.111**	**0.033**	**0.184**	**0.252**	**0.233**

Supplementary Discussion

Ascents: As in previous studies of frigatebirds[1-3], all of the birds in our study occasionally ascended to altitudes >600 m. These ascents occurred most often in the late afternoon (Supplementary Fig. 9b), suggesting that the birds were riding rising warm air currents[1-3]. The frigatebirds spent relatively little time at the peak altitude. Based on the accelerometer recordings, it appeared that the loss of altitude during the descent was due, in part, to feather preening. Although frigatebirds can glide in the air when they preen their body feathers, they drop abruptly when they draw their wings and flight feathers in toward the body for preening (Supplementary Movie 4).

While the focus of our study was not aimed at determining the function of these ascents, our findings have bearing on the question. Although sleep often occurred during high-altitude ascents, it did not occur during all ascents (Supplementary Movies 2 and 3). Moreover, sleep usually occurred at much lower altitudes (153.93±6.65 m, s.e.m.). Consequently, high-altitude ascents were not performed exclusively to facilitate sleep. As in raptors and storks migrating over land[4], frigatebirds may use rising air currents to gain height and thereby maximize displacement across the ocean as they glide down[3]. However, as shown in Supplementary Movie 3, after reaching the peak altitude the birds usually returned rapidly to lower altitudes. Also, as suggested by the accelerometry signals, the frigatebirds seemed to use the safety margin afforded by the high altitude to preen their flight feathers, a behavior that decreased displacement.

Common swifts (*Apus apus*) also spend the night flying and engage in high-altitude ascents[5]. Interestingly, common swifts ascend at precise times around dusk and dawn (see also[6]). The presence of the dawn ascent is particularly informative because it suggests that ascents serve a function other than simply providing an altitudinal buffer for the safe occurrence of sleep at

night, as previously proposed[7]. [Note: as stated in the introduction of the main text, sleep in flight has not been demonstrated previously in swifts or any other bird]. Instead, swifts may ascend to high altitudes at these times of the day to gain environmental information[5]. Although the timing of ascents in frigatebirds is far more variable than in swifts, in general terms, frigatebirds may also use ascents to assess the environment. For example, at high altitudes, frigatebirds may obtain a broader view of the ocean that facilitates the tracking of ocean eddies and associated favorable foraging conditions, a behavior that, like ascents, occurs throughout the day and night[8]. Frigatebirds ascending to high altitudes at night may also detect patches of bioluminescence predictive of high productivity and foraging opportunities. Finally, the proposed functions of ascents in frigatebirds are not mutually exclusive, and their relative importance may vary according to the time of day, environmental conditions, and phase (out-going or return) of the foraging flight.

REM sleep in flight? In all birds, periods of slow wave sleep (SWS) in flight were rarely interrupted by brief periods of low-amplitude, high-frequency EEG activity, associated with a gradual drop of the head, often punctuated by rapid twitches, as observed during REM sleep on land (Supplementary Fig. 13). At the end of these REM sleep episodes, the head was raised, and SWS usually resumed. Soaring and gliding continued uninterrupted by flaps or drops throughout these episodes.

Given the nature of avian REM sleep, its occurrence during flight may not be that surprising[9]. Notably, birds appear to have the ability to partially modulate muscle tone during REM sleep. For example, geese sleeping with their head supported on their back show mammalian-like neck muscle atonia during REM sleep, but when their head is facing forward and unsupported, partial muscle tone is maintained during REM sleep, resulting in a gradual, controlled drop, rather than a free fall of the head[10]. Similar mechanisms may also explain why birds are able to stand during

REM sleep. Consequently, frigatebirds may have an ability to maintain some tone during REM sleep in flight. When combined with the fact that episodes of REM sleep only last several seconds, any residual loss of muscle tone, as revealed in the accelerometer recordings, is unlikely to have an adverse effect on frigatebirds flying over the ocean.

Supplementary References

1. Weimerskirch, H., Chastel, O., Barbraud, C. & Tostain, O. Frigatebirds ride high on thermals. *Nature* **421**, 333–334 (2003).
2. De Monte, S. *et al*. Frigatebird behaviour at the ocean-atmosphere interface: integrating animal behaviour with multi-satellite data. *J. R. Soc. Interface* **9**, 3351–3358 (2012).
3. Weimerskirch, H., Bishop, C., Jeanniard du Dot, T., Prudor, A. & Sachs, G. Frigate birds track atmospheric conditions over months-long transoceanic flights. *Science* **353**, 74–78 (2016).
4. Akos, Z., Nagy, M. & Vicsek, T. Comparing bird and human soaring strategies. *Proc. Natl. Acad. Sci. U. S. A.* **105**, 139–4143 (2008).
5. Dokter, A. M. *et al*. Twilight ascents by common swifts, *Apus apus*, at dawn and dusk: acquisition of orientation cues? *Anim. Behav.* **85**, 545–552 (2013).
6. Liechti, F., Witvliet, W., Weber, R. & Bächler, E. First evidence of a 200-day non-stop flight in a bird. *Nat. Commun*. **4**, 2554 (2013).
7. Bäckman, J. & Alerstam, T. Confronting the winds: orientation and flight behaviour of roosting swifts, *Apus apus*. *Proc. Roy. Soc. B*, **268**, 1081–1087 (2001).
8. Tew Kai, E. *et al*. Top marine predators track Lagrangian coherent structures. *Proc. Natl. Acad. Sci. U. S. A.* **106**, 8245–8250 (2009).
9. Rattenborg, N. C. Do birds sleep in flight? *Naturwissenschaften* **93**, 413–425 (2006).

10. Dewasmes, G., Cohen-Adad, F., Koubi, H. & Le Maho, Y. Polygraphic and behavioral study of sleep in geese: existence of nuchal atonia during paradoxical sleep. *Physiol. Behav.* **35**, 67–73 (1985).
